# The Effect of Gold and Iron-Oxide Nanoparticles on Biofilm-Forming Pathogens

**DOI:** 10.1155/2013/272086

**Published:** 2013-09-25

**Authors:** Madhu Bala Sathyanarayanan, Reneta Balachandranath, Yuvasri Genji Srinivasulu, Sathish Kumar Kannaiyan, Guruprakash Subbiahdoss

**Affiliations:** ^1^Department of Biomedical Engineering, SSN College of Engineering, Old Mahabalipuram Road, Kalavakkam, Tamilnadu 603110, India; ^2^Department of Chemical Engineering, SSN College of Engineering, Old Mahabalipuram Road, Kalavakkam, Tamilnadu 603110, India

## Abstract

Microbial biofilms on biomaterial implants or devices are hard to eliminate by antibiotics due to their protection by exopolymeric substances that embed the organisms in a matrix, impenetrable for most antibiotics and immune-cells. Application of metals in their nanoparticulated form is currently considered to resolve bacterial infections. Gold and iron-oxide nanoparticles are widely used in different medical applications, but their utilisation to eradicate biofilms on biomaterials implants is novel. Here, we studied the effect of gold and iron oxide nanoparticles on *Staphylococcus aureus* and *Pseudomonas aeruginosa* biofilms. We report that biofilm growth was reduced at higher concentrations of gold and iron-oxide nanoparticles compared to absence of nanoparticles. Thus nanoparticles with appropriate concentration could show significant reduction in biofilm formation.

## 1. Introduction

In modern medicine, biomaterial implants and devices to support and restore functioning of body parts have become common with high success rates in terms of improved quality of life. A serious problem associated with the use of biomaterials is the occurrence of microbial infections. Biomaterial-associated infections (BAI), although of relatively low incidence, represent serious complications related to high mortality rates and high health care costs [1]. According to the studies conducted by European Centre for Disease Prevention and Control, the average prevalence of healthcare-associated infections in Europe was 7.1%; that is, approximately 4,131,000 patients were affected [2]. Moreover, costs related to healthcare-associated infection are greater than €7 billion in Europe annually [2]. A major proportion of the healthcare-associated infections and costs relate to BAI. To give an example, it has been reported that for approximately 800,000 artificial orthopaedic joints implanted in Europe, at least 1.5% will suffer from periprosthetic infections [2]. Whereas the costs of a primary implantation of an artificial hip or knee joint amount approximately €15.000, the costs of revision surgery due to infection easily triple [2]. With the average life-expectancy steadily increasing, concurrent with a nonnegotiable demand for a high quality of life, the use of biomaterials for the restoration of function will increase, including the incidence of BAI. In India, an estimation of 10% to 30% patients admitted to hospitals acquire nosocomial infection, and up to 70% of organisms causing infections are resistant to at least one antibiotic [3]. *Staphylococcus epidermidis* and *Staphylococcus aureus* are the most frequently isolated pathogens from infections related to biomaterials implant surfaces. In addition, isolated organisms include *Escherichia coli* and *Pseudomonas aeruginosa* [4].

Several biomaterial surface modifications [5–7] have been developed to reduce biofilm formation, but hitherto microbial adhesion can only be reduced by one or two log units and not fully eliminated. Thus long-term treatment of biofilms causing BAI remains a challenge [6].

Metals such as silver, copper, gold, titanium, and zinc have been used as antibacterial agents for centuries already [8], but their efficacy has been surpassed by modern antibiotics, and their use has diminished. Application of metals in their nanoparticulated form is currently considered to resolve bacterial infections, but has attracted scientific attention only over the past decade [8]. Nanoparticles are less than 100 nm in diameter, and as a result properties such as surface area, chemical reactivity, and biological activity alter dramatically. The antibacterial efficacy of metal nanoparticles has been suggested to be due to their high surface-to-volume ratio rather than to the sole effect of metal-ion release [8]. A high surface-to-volume ratio is generally accompanied by increased production of reactive oxygen species, including free radicals [9, 10]. These characteristics allow nanoparticles to interact closely with microbial membranes, damage their structure, and inactivate bacteria [8]. Metal oxide nanoparticles are of particular interest as antibacterial agents, as they can be prepared with extremely high surface areas and unusual crystalline morphologies with a high number of edges and corners, and other potentially reactive sites [11]. Therefore, the aim of this study was to evaluate the effect of gold and iron-oxide nanoparticles on biofilm-forming pathogens such as *S. aureus* and *P. aeruginosa*.

## 2. Materials and Methods

### 2.1. Synthesis

#### 2.1.1. Gold Nanoparticles

20 mL of hydrogen tetrachloroaurate and 2 mL of trisodium citrate dehydrate were added to a flask on a stirring hot plate. With continuous boiling at 100°C and stirring, gold chloride reduction by citrate is indicated by the change in colour from bluish purple to a deep red, indicating the formation of gold nanoparticles.

#### 2.1.2. Iron-Oxide Nanoparticles

4 mL of ferrous chloride and 1 mL of ferric chloride were added to a flask. Sodium hydroxide was added drop by drop and stirred continuously. Initially formed brown precipitate was changed into a black precipitate, indicating the formation of iron-oxide nanoparticles. The size of the synthesized particles was determined using transmission electron microscopy. The optical measurement of the nanoparticles was studied by UV-visible spectrophotometer (Unico) over the spectral range of 200–1000 nm.

### 2.2. Effect of Nanoparticles on Bacterial Biofilms

*Staphylococcus aureus* and *Pseudomonas aeruginosa* were used for this study.

Bacteria were first grown aerobically overnight at 37°C on blood agar from a frozen stock.

The plate was kept at 4°C. For each experiment, one colony was inoculated in 10 mL of tryptone soy broth (TSB) and cultured for 16 h. Bacteria were harvested by centrifugation.

The bacteria are suspended in TSB to a concentration of 10^6^ bacteria/mL.

Tissue culture polystyrene wells (12 wells) were filled with 1 mL of bacterial suspension and allowed to adhere and grow aerobically at 37°C for 4 h. Then, gold or iron-oxide nanoparticles were introduced in different concentrations (0.01 mg/mL, 0.05 mg/mL, 0.10 mg/mL, and 0.15 mg/mL). Thereafter, biofilms were allowed to grow for 24 h. Subsequently, wells were washed with sterile water to remove unbound bacteria, and biofilm development was assessed by measuring the optical density (absorbance at 590 nm) using a spectrophotometer. Data are presented as percentage change in biofilm growth in the presence of nanoparticles with respect to absence of nanoparticles (control). Experiments were performed in triplicate with separately cultured bacteria.

### 2.3. Statistical Analysis

Experiments were performed in triplicate. Data are represented as a mean with standard deviation. For statistical analysis ANOVA was performed followed by a Tukey's HSD post hoc test, and a *P* value < 0.05 was considered to be significant.

## 3. Results and Discussion

**Figure 1:**
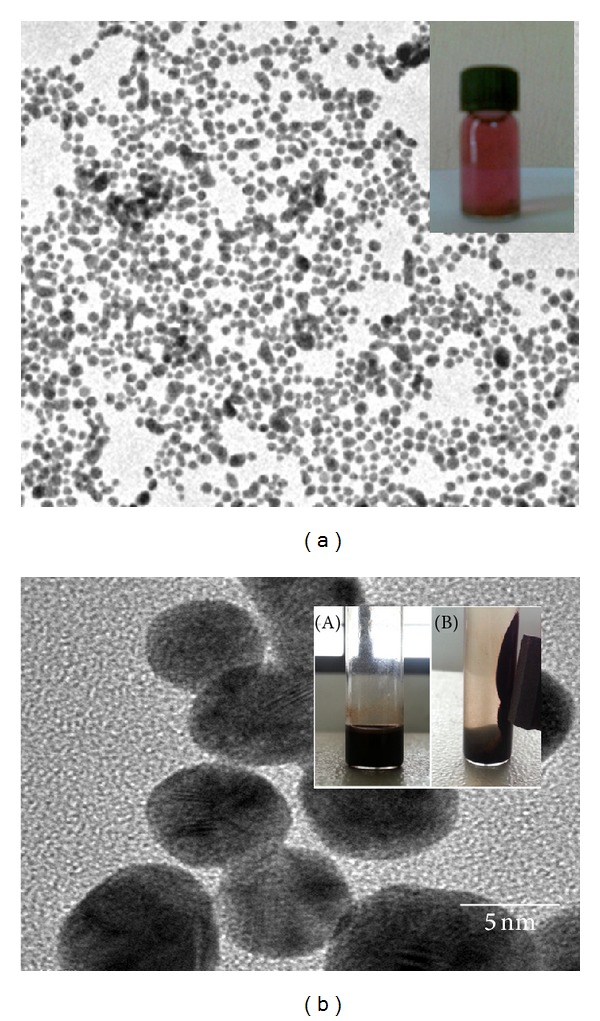
Transmission electron micrographs of (a) gold and (b) iron-oxide nanoparticles. Inset images show the synthesized nanoparticles. (b)(B) shows the magnetic property of iron-oxide nanoparticles. Bar denotes 5 nm.

The TEM images of synthesized gold and iron-oxide nanoparticles are shown in Figure 1. The nanoparticles were measured less than 10 nm.

**Figure 2:**
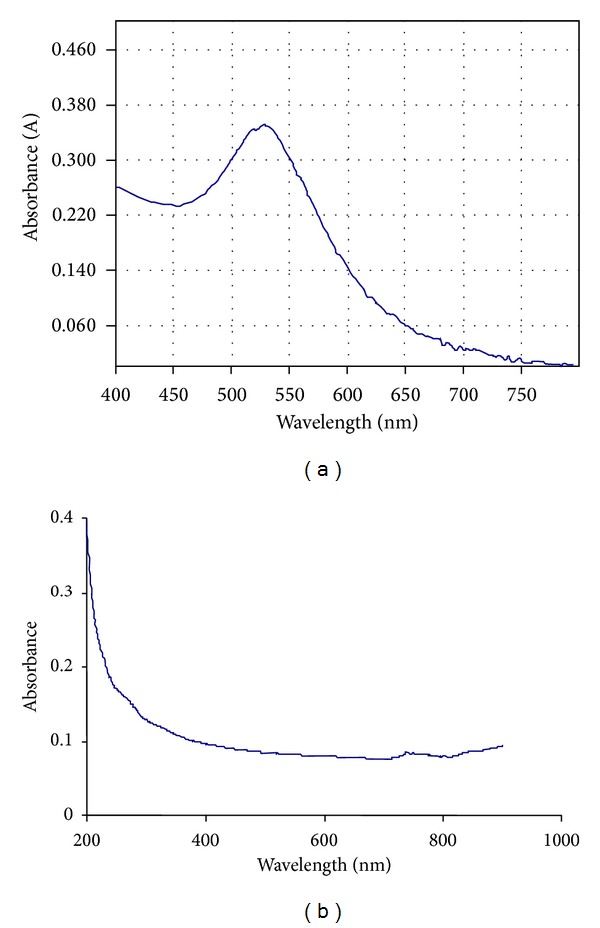
UV-visible spectrum of (a) gold and (b) iron-oxide nanoparticles.

**Figure 3:**
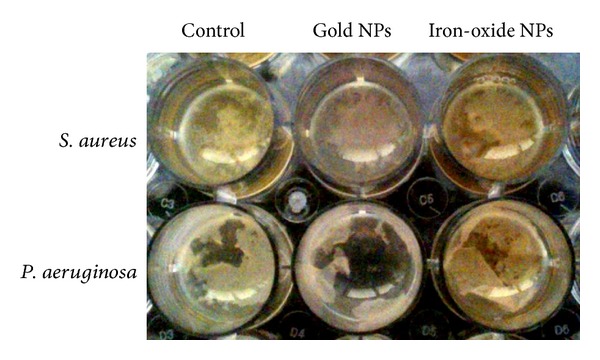
Biofilm formation of *S. aureus* and *P. aeruginosa* after 24 h of growth in the presence of gold and iron-oxide nanoparticles (0.01 mg/mL).

The UV-visible spectrum of citrate-stabilized gold nanoparticles was shown in Figure 2(a). The plasmon band observed for the wine-red colloidal gold at 518 nm is the characteristics of gold nanoparticles. The UV-visible spectrum of iron-oxide nanoparticles was shown in Figure 2(b) where the absorbance of nanoparticles steadily decreases with time which confirms the formation of oleic acid-coated iron-oxide nanoparticles. Bacterial biofilm growth after 24 h in the presence of gold and iron-oxide nanoparticles was shown in Figure 3.

**Figure 4:**
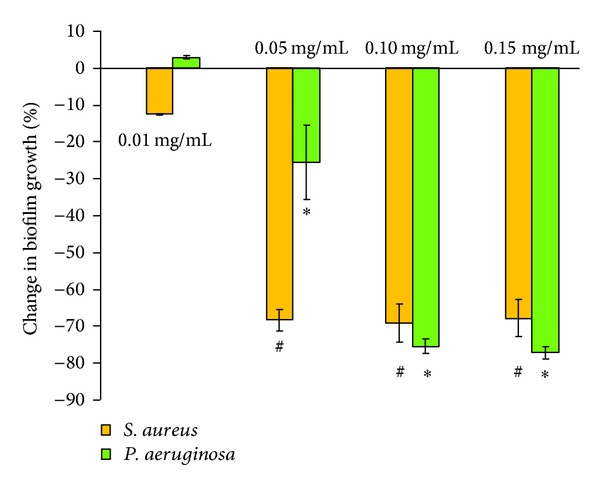
Percentage change in biofilm growth in the presence of gold nanoparticles (0.01 mg/mL, 0.05 mg/mL, 0.10 mg/mL, and 0.15 mg/mL) with respect to biofilm growth in the absence of nanoparticles (control). Error bar represents the standard deviations over three replicates, with separately cultured bacteria. #, ∗ denote significance at differences at *P* < 0.01 compared with 0.01 mg/mL.

**Figure 5:**
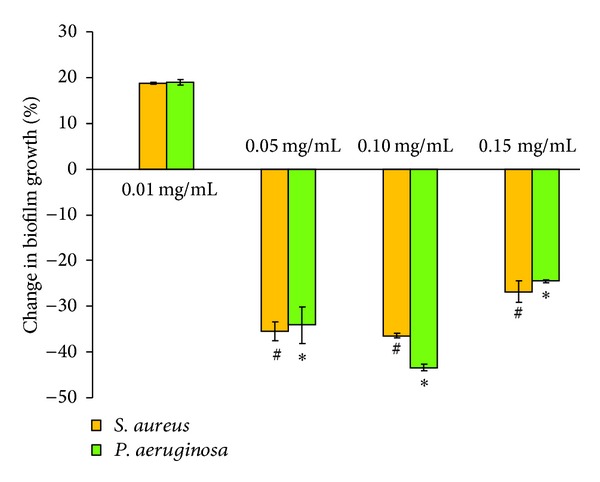
Percentage change in biofilm growth in the presence of iron-oxide nanoparticles (0.01 mg/mL, 0.05 mg/mL, 0.10 mg/mL, and 0.15 mg/mL) with respect to biofilm growth in the absence of nanoparticles (control). Error bar represents the standard deviations over three replicates, with separately cultured bacteria. #, ∗ denote significance at differences at *P* < 0.01 compared with 0.01 mg/mL.

*In vitro* experimental results showed significant effect in bacterial biofilm growth in the presence of gold and iron-oxide nanoparticles. Differences were observed with respect to the type of bacteria, nanoparticle, and nanoparticle concentrations. At a concentration of 0.01 mg/mL, gold nanoparticles showed reduction (−13%) in *S. aureus* biofilm growth, whereas iron-oxide nanoparticles showed increased (19%) *S. aureus* biofilm growth compared to control (OD = 1.05). Interestingly, *P. aeruginosa* biofilm growth was increased in the presence of gold and iron-oxide nanoparticles (0.01 mg/mL) compared to control (OD = 1.31). However, at higher concentrations (0.05 mg/mL, 0.10 mg/mL, and 0.15 mg/mL) a significant reduction in biofilm growth (*S. aureus* and *P. aeruginosa*) was observed in the presence of gold (Figure 4) and iron-oxide (Figure 5) nanoparticles compared to low concentration (0.01 mg/mL). Similarly, Taylor and Webster [12] showed that iron-oxide nanoparticles in a concentration range of 0.01 to 2 mg/mL were able to kill up to 25% of *S. epidermidis* in a 48 h old biofilm. And, similar results were observed in our previous study with iron-oxide nanoparticles on *S. aureus* biofilms [13]. In contrast, Haney et al. [14] showed an increase in *P. aeruginosa* biofilm biomass in the presence of 0.2 mg/mL of superparamagnetic iron-oxide nanoparticles. In the presence of gold nanoparticles, Zhang et al. [15] showed a reduction of 64% in viable bacteria. Similarly, another study demonstrated that gold nanoparticles showed excellent antibacterial potential against gram-negative bacteria *Escherichia coli* and gram-positive bacteria Bacillus Calmette-Guerin. This study also proposed that the antibacterial activity could be due to uptake of single gold nanoparticles by bacteria and rearrangement of them inside cytoplasm [16].

The antibacterial activity of iron-oxide nanoparticles could be due to several mechanisms. The main mechanism suggested is related to oxidative stress generated by ROS [17]. ROS includes superoxide radicals, hydroxyl radicals, hydrogen peroxide, and singlet oxygen that may cause chemical damage to proteins and DNA in bacteria [18]. Secondly, electrostatic interactions between nanoparticles and bacterial cell membranes or cell membrane proteins can result in physical damage, which ultimately leads to bacterial cell death [17]. Other studies demonstrated that the small size of nanoparticles could contribute to their antibacterial effects [19, 20]. Lee et al. [18] reported that inactivation of *E. coli* could be due to the penetration of nanoparticles with sizes ranging from 10 to 80 nm into *E. coli* membranes.

## 4. Conclusion

Recent developments in the field of nanoparticles [8, 12, 18] and promising results from *in vitro* studies provide for a new and urgently needed strategy for the treatment of biomaterial-implant-associated infections using nanoparticles.
